# Maternal siRNA silencing of placental SAA2 mitigates preterm birth following intrauterine inflammation

**DOI:** 10.3389/fimmu.2022.902096

**Published:** 2022-09-23

**Authors:** Yang Liu, Jin Liu, Anguo Liu, Hillary Yin, Irina Burd, Jun Lei

**Affiliations:** ^1^ Integrated Research Center for Fetal Medicine, Department of Gynecology and Obstetrics, Johns Hopkins University School of Medicine, Baltimore, MD, United States; ^2^ Department of Obstetrics and Gynecology, The Second Xiangya Hospital of Central South University, Changsha, China

**Keywords:** preterm birth, intrauterine inflammation, serum amyloid A (SAA), placenta, siRNA treatment

## Abstract

The placental inflammatory processes induced maternally result in preterm birth (PTB). Serum amyloid A (SAA) is a well-known biomarker of inflammation. The objective of this study was to investigate whether murine placental SAA isoforms (SAA1–4) participate in the mechanism of spontaneous PTB and whether maternal regulation of SAA production may serve as a therapeutic approach. During the gestation, all isoforms of SAA were detectable except SAA2. The mouse model of intrauterine inflammation was established using LPS infusion to the uterus. Following intrauterine inflammation, placental SAA2 increased significantly. Inhibition of *Saa2*, using si*Saa2*, markedly decreased PTB. The increased placental expression of pro-inflammatory cytokines *Il1β*, *Il6*, and *Tnfα* were downregulated by si*Saa2* treatment. Maternal inhibition of *Saa2* did not change the expression of *Saa1–4* in the fetal brain. Explant inflammatory culture of placentas with si*Saa2* showed similar results to our *in vivo* experiments. This study demonstrates the highly expressed placental SAA2 as a novel therapeutic target, and maternal administration of siRNA as a promising approach to alleviate PTB.

## Introduction

Preterm birth (PTB), defined as parturition occurring prior to 37 weeks of gestation, accounts for over 50% of all neonatal deaths worldwide (1–3). An estimated 15 million babies are born preterm every year, and this number is rising (4). While medical technologies for supporting the survival of preterm infants have become more effective, babies may face a lifetime of disabilities, including chronic lung and gastrointestinal difficulties, cerebral palsy, developmental delay, and vision and hearing deficits (5–7). To date, only treatments with limited efficacy or restrictive safety are available (8).

With multiple factors contributing to PTB, intrauterine inflammation is thought to play a key role (9, 10). Defining key candidates from the inflammation-induced immune cascade may provide novel targets and assist in building up preventive strategies. Serum amyloid A (SAA) is a well-known biomarker of inflammation (11). Local expression of SAA is detected in the placenta at a comparable level to other tissues (12–14). High levels of serum SAA have been reported in correlation with premature delivery (15–18). SAA is induced by various pro-inflammatory cytokines, such as interleukin-1β (IL-1β), IL-6, and tumor necrosis factor α (TNF-α) (19–21). Biologically, SAA appears to have a broad range of activities mediated by various receptors, suggesting not only as a biomarker but also to be an integral center of inflammatory signals (22, 23).

The murine SAA gene family is composed of five members (*Saa1–5*) (24), including one (*Saa5*) pseudogene; similarly, the human SAA gene family is composed of four members (*SAA1–4*), including one (*SAA3*) pseudogene (25, 26). A few clinical studies have shown that SAA1/2 in cord blood increases in preterm infants and unexplained recurrent early pregnancy losses (16, 18, 27). Intraperitoneal injection of recombinant SAA1 not only induces PTB but also increases the placental cytokine expression in mice (28). Another study reported that administration of a truncated SAA1 fragment reduces the severity of acute lipopolysaccharide (LPS)-challenged lung injury in mice (29). However, the gestational course in the expression of placental SAA isotypes is missing, and whether and how these isotypes participate in the occurrence of PTB remains unknown. This information would be instrumental in designing novel approaches for PTB.

RNA interference (RNAi) is a naturally occurring endogenous regulatory process (30). Using synthetic small interfering RNA (siRNA), RNAi represents the tremendous therapeutic potential that aims at disease-causing genes, leading to decreased abundance of the corresponding protein (31). This promising therapeutic platform enables more precise and personalized treatment (32). A remarkable progress has been achieved that siRNA therapeutics was approved by FDA for clinics (31–33). However, considering the application of siRNA treatment to obstetrics diseases including PTB, studies on efficacy and safety are the primary to indispensably take into account.

In this study, we hypothesized that one or more SAA isoforms in the placenta serves a crucial role in the maternal inflammation-induced PTB, and maternal administration of siRNA against *Saa* decreases PTB. The aim of this study was to explore changes of SAA isoforms in the placenta, maternal liver, and fetal brain, and to evaluate the effect of maternal siRNA against *Saa* (si*Saa*) on PTB, fetal viability, and pro-inflammatory cytokines.

## Materials and methods

### Ethics statement

This study was carried out in accordance with the recommendations in the Guide for the Care and Use of Laboratory Animals of the National Institutes of Health. The protocols were approved by the Institutional Animal Care and Use Committee and Health Safety and the Environment at Johns Hopkins University.

### Animal preparation

Pregnant CD-1 dams (Charles River Laboratories, Wilmington, MA, USA) were used for the study. To evaluate the developmental expression of SAA proteins in the placenta, four dams were sacrificed daily from embryonic days (E) 10 to 18 (n = 36). The mouse model of intrauterine inflammation was established as previously described (34–36). Briefly, dams on E17 were placed under isoflurane anesthesia (Baxter Healthcare, Deerfield, IL, USA) following a mini-laparotomy. The dose of 25 μg of LPS (E. coli O55:B5, Sigma-Aldrich, St Louis, MO, USA, n = 94) in 100 μl PBS or vehicle only (PBS, n=61) was injected between the first and second embryos of the lower right uterine horn. Routine laparotomy closure was performed, and dams were returned to cages individually. One hour after surgery, dams were randomly allocated to the treatment of SAA intraperitoneal (IP) injection: SAA1 (amino acids (aa) 11–58, GenScript, Piscataway, NJ, USA, n = 20) at 5 mg/kg or SAA2 (aa 20–122, R&D Systems, Minneapolis, MN, USA, n = 25) at 40 µg/kg in 100 µl PBS. The concentrations were determined based on methods published previously (29) (37).

Another set of dams were applied to siRNA treatment 1 h after intrauterine inflammation. si*Saa2* (Thermo Fisher Scientific, Halethorpe, MD, USA, n = 10) at a dose of 74 nmol/kg in 100 µl PBS was injected intravenously (IV) though the tail vein. Transfection reagent alone (siNC) into PBS- or LPS-infused mice served as our transfection control. PTB and fetal viability were observed. Fetal viability was defined as the percentage of viable fetuses within the inoculated uterine horn. N = total number of fetuses from dams per group. Aborted fetuses were determined by the following characteristics: pale skin, lacking responses, small body size, and no breathing. The placenta, maternal liver, and fetal brain were harvested 24 h post injection (hpi), either fixed in 4% paraformaldehyde (PFA) overnight or immediately fresh frozen at -80°C.

### Western blot

Frozen tissues were homogenized on ice in RIPA lysis buffer (Sigma‐Aldrich, St, Louis, MO, USA) with proteinase inhibitor (Sigma‐Aldrich) and phosphatase inhibitor cocktail 2 (Sigma‐Aldrich). The homogenized specimens were then placed on ice for 15 min and centrifuged at 14,000 rpm for 20 min at 4°C. The resulting supernatants were collected for further experiments. Total protein was separated by sodium dodecyl sulfate‐polyacrylamide gel electrophoresis (SDS‐PAGE, Bio‐Rad, Hercules, CA, USA) using 4%–15% gels (Bio‐Rad) and then transferred onto nitrocellulose membranes (Bio‐Rad) using a semidry transfer device (Trans‐Blot^®^ Turbo™, Bio‐Rad). Membranes were blocked with 5% bovine serum albumin (BSA, Sigma‐Aldrich) in Tris‐buffered saline (Corning, New York, NY, USA) plus 0.1% Tween‐20 (Sigma‐Aldrich) (TBST) for 30 min at room temperature and incubated with primary antibodies in 5% of BSA at 4°C overnight, then washed with TBST. The following primary antibodies were purchased: rabbit anti-SAA1 antibody (Abcam, Cambridge, MA, USA), rabbit anti-SAA1 antibody (LSBio, Seattle, WA, USA), rabbit anti-SAA2 antibody (Biomatik, Wilmington, DE, USA), rabbit anti-SAA2 antibody (LSBio), rabbit anti-SAA3 (MyBioSource, San Diego, CA, USA), rabbit anti-SAA3 (Abcam), and rabbit anti-SAA4 (MyBioSource). Rabbit anti-actin (Abcam) or Rabbit anti-GAPDH (Abcam) was used as the quantitation control. Goat anti‐rabbit IR Dye‐800CW (Li‐Cor, Lincoln, NE, USA) was used as the secondary antibody for all primary antibodies. Image acquisition was performed using Li‐Cor Odyssey Near Infra‐Red System (LI-COR Biosciences, Lincoln, NE, USA). ImageJ software (v1.48, National Institutes of Health, Bethesda, MD, USA) was used to analyze the protein bands of targets.

### Real-time quantitative polymerase chain reaction

Total RNA was extracted from fresh frozen tissues with RNeasy Mini Kit (Qiagen, Germantown, MD, USA). Two micrograms of RNA was applied for complementary (c) DNA synthesis in a 40-µl reaction, using Bio‐Rad iScript™ cDNA Synthesis Kit (Bio‐Rad). The primers for *Saa*1 (Mm.PT.58.210905888), *Saa*2 (Mm.PT.58.43376774), *Saa*3 (Mm.PT.58.10949783), *Saa*4 (Mm.PT.58.41916253), *Il6* (Mm.PT.58.10005566), *Tnfα* (Mm.PT.58.12575861), *Il1β* (Mm.PT.58.41616450), and *β‐actin* (Mm.PT.39a.22214843) were obtained from Integrated DNA Technologies (Coralville, IA, USA). The primer for *18S* (Cat. No. 4310893E) was obtained from Applied Biosystems (Thermo Fisher Scientific). Real-time quantitative polymerase chain reaction (RT-qPCR) was performed with Universal Master Mix II (Life Technologies, Frederick, MD, USA) on a CFX384 Real-Time PCR Detection System (Bio-Rad). Transcript levels were determined by normalizing the target gene threshold cycle (CT) value to the CT value of the endogenous housekeeping gene *β‐actin* and *18S* (ΔCT).

### Immunohistochemistry

PFA-fixed placentas were rinsed with PBS followed by storage in 30% sucrose (Sigma‐Aldrich) for cryoprotection. Using a cryostat (Leica; Buffalo Grove, IL), placentas were cut into sections of 20-μm thickness and further incubated overnight at 4°C with rabbit anti-SAA2 antibody (Biomatik) or rat anti-CD45 (Thermo Fisher Scientific) in PBS containing 0.5% Triton X-100 (Sigma-Aldrich). The next day, the sections were rinsed with PBS and then incubated with fluorescent goat anti-rabbit DyLight 568 (Abcam) or goat anti-rat DyLight 488 (Abcam) secondary antibody for 3 h at room temperature. The sections were further stained with diamidino-2-phenylindole (DAPI, Roche, Indianapolis, IN, USA) for 2 min at room temperature followed by mounting with Fluoromount-G (eBioscience, San Diego, CA, USA). Images were obtained using an Axioplan 2 Imaging system (Carl Zeiss, Thornwood, NY, USA) from the same staining batch.

### Primary explant culture

On E17, placentas were removed and briefly placed in ice-cold sterilized PBS. Explant cultures were performed as previously described (38–40). Briefly, the placenta was cut into two pieces and was then placed into individual wells of six-well plates containing 2 ml of the tissue culture media (45% DMEM/45% Ham’s F12/10% FBS + pen/strep) and incubated at 37°C with a sterile gas mixture containing 8% O_2_, 5% CO_2_, and 87% N_2_ for 24 h. Explants were cultured with the addition of various reagents as follows: LPS (100, 500, 1000 ng/ml) and/or si*Saa2* (0.56 nmol/ml, Thermo Fisher Scientific). PBS+si*Saa2* was applied as our control. Time “zero” was the time of plating. Media and explant tissues were collected and stored at −80°C until use.

### Cell cytotoxicity by lactate dehydrogenase assay

Cell cytotoxicity was determined by measuring lactate dehydrogenase (LDH) activity in culture media using a commercially available kit (Sigma-Aldrich). Briefly, the media were centrifuged at 3,000 rpm for 5 min. Cell-free culture supernatants were then incubated with the assay buffer and substrate mix in a new plate at room temperature. The absorbance at 490 nm was measured using a 96-well microplate reader (CLARIOstar BMG LABTECH, Cary, NC, USA). The background (spontaneous LDH release) value was set by measuring the media at time zero start from plating. Experiments were performed in triplicates.

### Flow cytometry

Single cells from explants or *in vivo* placentas were prepared by manual and enzymatic (collagenase D, Roche, Indianapolis, IN, USA) digestion followed by passing through a 70‐μm nylon mesh (BD Falcon cell strainer). Red blood cell lysis (ACK lysing buffer, Thermo Fisher Scientific) and total cell counts were performed followed by staining of a surface marker for leukocytes and anti-mouse CD45 (clone 30-F11, Thermo Fisher Scientific). The staining was incubated in fluorescence-activated cell sorting (FACS) buffer with 1 mM EDTA (Quality Biological, Gaithersburg, MD, USA) for 30 min at 4°C in the dark. OneComp eBeads (eBioscience) were used for compensation. Data were acquired on an Attune™ NxT Acoustic Focusing Cytometer (Thermo Fisher Scientific) and analyzed with FlowJo version 10.1 (FlowJo, LLC, Ashland, OR, USA). Debris and doublets were excluded by sequential gating on forward scatter height versus forward scatter area followed by gating on CD45+ leukocytes.

### Enzyme-linked immunosorbent assay

Measurements of IL-1β and TNF-α secreted into cultural media of placental explants were performed using IL-1β and TNF-α mouse enzyme-linked immunosorbent assay (ELISA) kits (Abcam), respectively, according to the protocol provided by the manufacturer. Experiments were performed in triplicates.

### Terminal deoxynucleotidyl transferase dUTP nick end labeling assay

Placental sections were stained using the Click-iT™ Plus TUNEL Assay kit (Thermo Fisher Scientific) following the manufacturer’s protocol. The terminal deoxynucleotidyl transferase dUTP nick end labeling (TUNEL)-positive apoptotic cells were counted using an Axioplan 2 Imaging system.

### Statistical analyses

Data analyses were performed using GraphPad Prism 8 (GraphPad Software, La Jolla, CA, USA). Grubbs’ test for outliers was conducted for each experimental group, and noted outliers were removed prior to analysis. PTB and abortion were analyzed using the chi-square test. Data from Western blot and RT-qPCR were analyzed using one-way ANOVA. Between comparable groups, Bonferroni *post-hoc* test was used for comparisons of normally distributed data and Kruskal–Wallis with Dunn’s comparisons were for non-parametric data. A *p* value <.05 was considered significant.

## Results

### Placental SAA2 is undetectable during middle and late gestation

**Figure 1:**
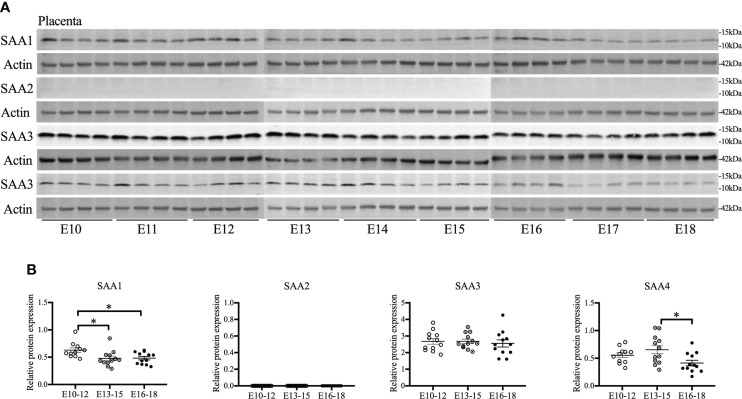
The gestational course of serum amyloid A (SAA) isoforms in the placenta. One placenta was harvested daily from each CD-1 dam starting from embryonic (E) day 10 and ending on E18. Western blot was performed to detect the expression of SAA isoforms. **(A)** Gel images of SAA1–4 expression. **(B)** Statistical analysis of SAA1–4 expression normalized to the levels of actin as a loading control. n = 4 dams for each day. Values are expressed as mean ± SEM, *p < 0.05 by one-way ANOVA.

As placentas form on E10.5 (41), the gestational course of SAA1–4 expression in placentas from middle to late gestation (E10–18) was evaluated. SAA1 expression was significantly decreased on E13–15 compared with E10–12 (*p* < 0.05, **Figures 1A, B** **)** and maintained at the same level until E16–18. SAA2 expression was undetectable at all the time points examined (**Figures 1A, B** **).** A further confirmatory experiment was performed using another commercial SAA1–3 antibodies **(** **Supplemental Figure 1**). There were no significant changes in SAA3 expression during the whole middle and late gestation (**Figures 1A, B**). Alternatively, SAA4 expression was reduced significantly on E16–18 compared with E13–15 (*p* < 0.05, **Figures 1A, B** **)**. Our data suggest that each isoform of SAA in the placenta displays differential expression patterns across the gestation. Notably, endogenous placental SAA2 is absent from E10 to 18 examined.

### Placental SAA2 increases following intrauterine inflammation

**Figure 2:**
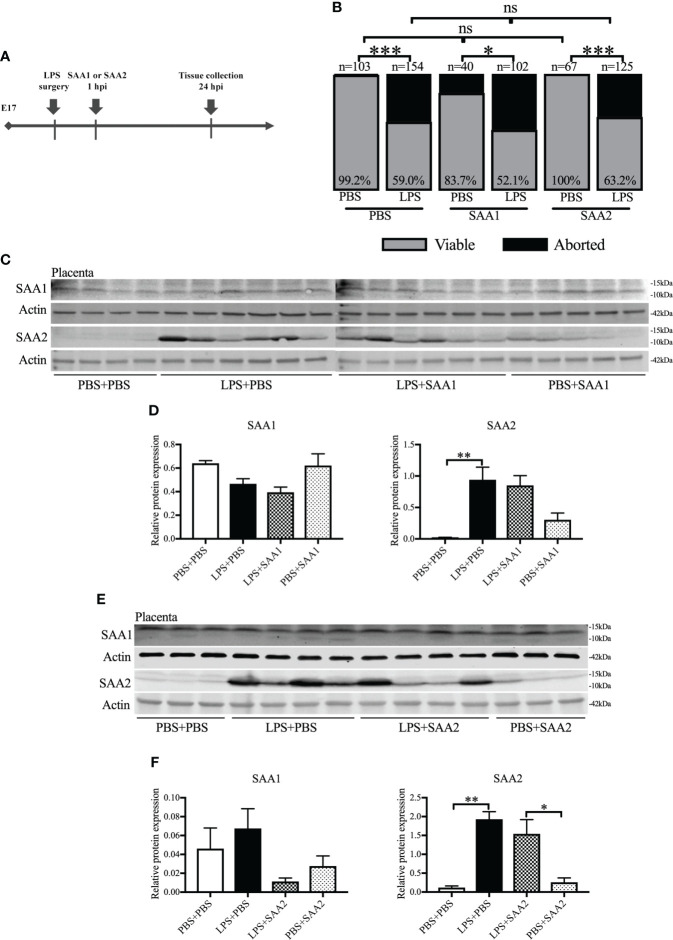
The expression of serum amyloid A (SAA) 2 in the placenta increases following intrauterine inflammation. **(A)** Study strategy. On embryonic (E) day 17, CD-1 mice underwent a mini-laparotomy in the lower abdomen for intrauterine injection of lipopolysaccharide (LPS). One hour later, dams received intraperitoneal injection of SAA1 or 2 fragment or phosphate-buffered saline (PBS). At 24 h postinjection (hpi), dams were sacrificed, and fetal viability was determined as the percentage of fetuses within the injected uterine horn that were viable. Western blot was performed. **(B)** The fetal viability per litter was determined. PBS+PBS, n = 103 from 15 dams; LPS+PBS, n = 154 from 20 dams; PBS+SAA1, n = 40 from eight dams; LPS+SAA1, n = 102 from 12 dams; PBS+SAA2, n = 67 from 10 dams; LPS+SAA2, n = 125 from 18 dams. **(C, D)** Gel images of SAA1 and SAA2 expression in the placenta and statistical analysis normalized to the levels of actin as a loading control with SAA1 injection. PBS+PBS, n = 4; LPS+PBS, n = 6; LPS+SAA1, n = 6; PBS+SAA1, n = 5. **(E, F)** Gel images of SAA1 and SAA2 expression in the placenta and statistical analysis normalized to the levels of Actin as a loading control with SAA2 injection. PBS+PBS, n = 3; LPS+PBS, n = 4; LPS+SAA2, n = 4; PBS+SAA2, n = 3. Values are expressed as mean ± SEM. **(B)** Chi-square test. ns, not significant. **(D)** and **(F)** One-way ANOVA. *p < 0.05, **p < 0.01, ***p < 0.001.

SAA1 and SAA2 are generally acknowledged as inflammation biomarkers (26). We examined the expression of SAA isoforms following intrauterine inflammation. To identify the role of SAA1 or SAA2, we IP injected SAA1 or SAA2 fragments alone or with LPS into dams on E17 (**Figure 2A**). Similar to our previous study (42), there was markedly increased abortion at 24 hpi and PTB in LPS+PBS compared with the control (PBS+PBS, *p* < 0.05, **Figure 2B** and **Table 1**). SAA1/2 alone (PBS+SAA1/2) did not induce abortion or PTB, i.e., no significant effect was observed compared with PBS+PBS (*p* > 0.05, **Figure 2B** and **Table 1** **).** Moreover, increased abortion was not observed in the LPS+SAA1/2 group compared with LPS+PBS (*p* > 0.05, **Figure 2B** and **Table 1**), implying that SAA1/2 at the presently studied dose was not sufficient to induce abortion or exacerbate LPS-induced abortion.

**Table 1:** Preterm birth rate.

Group	Preterm birth rate
PBS+PBS (n = 27)	0%
LPS+PBS (n = 34)	44.1%^*^
PBS+SAA1 (n = 8)	0%
LPS+SAA1 (n = 12)	33.3%^#^
PBS+SAA2 (n = 10)	0%
LPS+SAA2 (n = 18)	33.3%^§^
PBS+si*Saa2* (n = 6)	0%
LPS+si*Saa2* (n = 18)	11.1%^†^
PBS+siNC (n = 10)	0%
LPS+siNC (n= 12)	33.3% ^‡^

*p < 0.05, compared with PBS+PBS; # p < 0.05, compared with PBS+SAA1;

^§^p < 0.05, compared with PBS+SAA2; † p < 0.05, compared with LPS+PBS;

^‡^p < 0.05, compared with PBS+siNC. Chi-square test. NC, negative control.

Western blot was further utilized to analyze the changes of SAA isoforms in the placenta and maternal liver. Liver is usually recognized as the primary resource of SAAs (43). At 24 hpi, only SAA2 was significantly increased, more than 10–40 times higher in LPS+PBS compared with PBS+PBS (*p* < 0.01, 0.941 ± 0.2 *vs*. 0.020 ± 0.01, **Figures 2C, D**; 1.930 ± 0.200 *vs*. 0.119 ± 0.044, **Figures 2E, F**). There were no significant changes in SAA1 (*p* > 0.05, **Figures 2C–F** **)**, 3, and 4 expression (*p* > 0.05, **Supplemental Figures 2A, B**) in the placenta following intrauterine inflammation (LPS+PBS) compared with PBS+PBS. Maternal administration of SAA1 or 2 alone (PBS+SAA1 or PBS+SAA2) or following intrauterine inflammation (LPS+SAA1 or LPS+SAA2) did not affect SAA1–4 expression in the placenta compared with PBS+PBS and LPS+PBS, respectively (*p* > 0.05, **Figures 2C–F**; **Supplemental Figures 2A, B**). In the maternal liver, there was an increased trend in SAA2 expression in LPS+PBS (3.587 ± 0.251), compared with PBS+PBS (2.092 ± 0.203, **Supplemental Figures 2C, D**). Taken together, these data illustrate that placental SAA2, but not SAA1, 3, or 4, is very susceptible to intrauterine inflammation.

### Maternal administration of siSaa2 alleviates abortion and preterm birth induced by intrauterine inflammation

**Figure 3:**
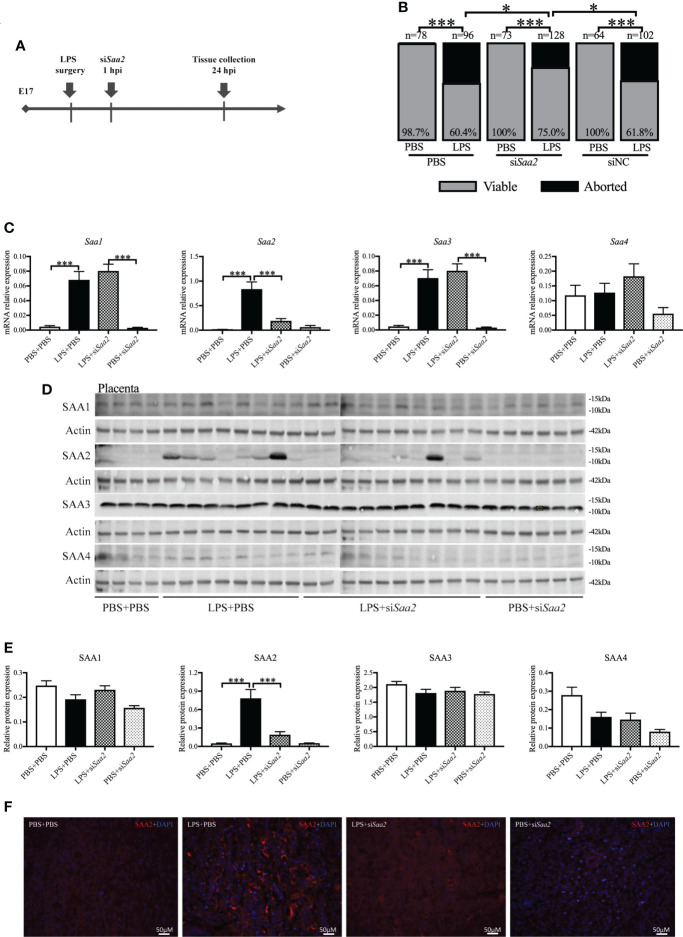
Maternal administration of siRNA against *Saa2* alleviates abortion induced by intrauterine inflammation. **(A)** Study strategy. On embryonic (E) day 17, CD-1 mice underwent a mini-laparotomy in the lower abdomen for intrauterine injection of lipopolysaccharide (LPS). One hour later, dams received intravenous injection of siRNA against serum amyloid A 2 (si*Saa2*) or phosphate-buffered saline (PBS). At 24 h postinjection (hpi) dams were sacrificed, and fetal viability was determined as the percentage of fetuses within the injected uterine horn that were viable. **(B)** The fetal viability per litter was determined. PBS+PBS, n = 78 from 12 dams; LPS+PBS, n = 96 from 14 dams; PBS+si*Saa2*, n = 40 from six dams. LPS+si*Saa2*, n = 128 from 18 dams; PBS+siNC, n = 64 from 10 dams, LPS+siNC, n = 102 from 12 dams. NC, negative control. **(C)** One placenta per dam was harvested, and real time-quantitative polymerase chain reaction (RT-qPCR) was performed to evaluate the effect of siRNA treatment. **(D)** Gel images of SAA1-4 expression in the placenta using Western blot. **(E)** Statistical analysis of placental SAA1–4 expression normalized to the levels of actin as a loading control. PBS+PBS, n = 6; LPS+PBS, n = 10; LPS+si*Saa2*, n = 10; PBS+si*Saa2*, n = 6. Values are expressed as mean ± SEM, **(B)** chi-square test. **(C, E)** One-way ANOVA. *p < 0.05, ***p < 0.001. **(F)** Representative fluorescence immunostaining for SAA2 (red) and DAPI (blue, nucleic staining) in the labyrinth of placentas. Scale bars: 50 μm.

To explore whether placental SAA2 not only functions as a biomarker but also participates in the mechanism of adverse fetal outcomes induced by intrauterine inflammation, another set of experiments were performed to maternally inhibit SAA2 expression using si*Saa2* (**Figure 3A**). Treatment with si*Saa2* (LPS+si*Saa2*) markedly increased the viability from 60.4% to 75.0% (*p* < 0.05, **Figure 3B**) and decreased PTB from 44.1% to 11.1% (*p* < 0.05, **Table 1**) compared with LPS+PBS.

RT-qPCR, Western blot and immunohistochemistry (IHC) were performed to verify the effect of maternal si*Saa2* administration in the placenta and maternal liver. At 24 hpi, in LPS+PBS, mRNA levels of placental *Saa1*, *Saa2*, and *Saa3* were significantly elevated (*p* < 0.001, **Figure 3C**) compared with PBS+PBS. Administration of si*Saa2* markedly reduced *Saa2* expression in the placenta (*p* < 0.001, **Figure 3C**) without altering *Saa1*, *Saa3*, and *Saa4* (**Figure 3C**). There was no significant difference between PBS+si*Saa2* and PBS+PBS for *Saa1–4* (*p* > 0.05, **Figure 3C** *)*. At the protein level, in the placenta, increased SAA2 expression induced by LPS was significantly decreased by si*Saa2* treatment (*p* < 0.001, **Figures 3D, E**). The expression of SAA1, 3, and 4 was not changed by either LPS or additional si*Saa2* infusion (*p* > 0.05, **Figures 3D, E**). IHC of placental SAA2 showed the similar expression pattern to Western blot (**Figure 3F**). In the maternal liver, it appeared that SAA2 expression was in a decreased trend by si*Saa2* administration (LPS+si*Saa2*) compared with LPS+PBS, but not reaching significance (**Supplemental Figures 3A, B** **)**. The other isoforms were not changed by either LPS or si*Saa2* treatment (**Supplemental Figures 3A, B** **)**. Our data indicate that maternal delivery of siRNA against *Saa2* effectively inhibits SAA2 expression in the placenta without affecting other isoforms of maternal SAA.

### SAA2 expression increases in placental explants following inflammation in a dose-dependent manner

**Figure 4:**
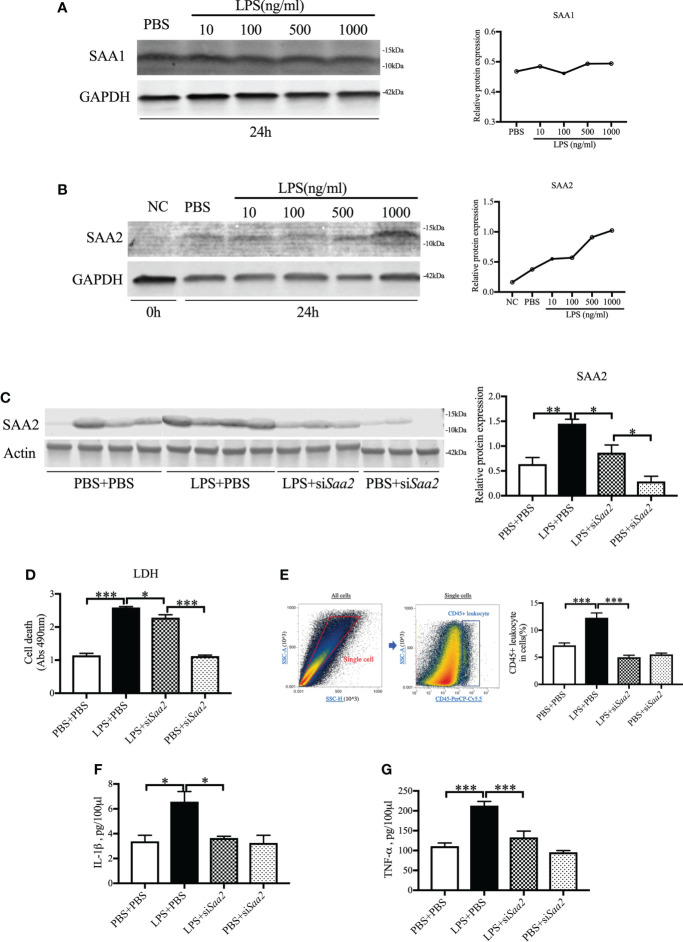
siRNA against *Saa2* reduces SAA2 and decreases inflammation in explant culture of placental tissue following LPS exposure. On E17, placenta slices were stimulated with control media and lipopolysaccharide (LPS 100, 500, 1,000 ng/ml) with or without siRNA against serum amyloid A 2 (si*Saa2*) for 24 (h) Gel images of SAA1 **(A)** and SAA2 **(B)** expression and statistical analysis normalized to the levels of GAPDH as a loading control. **(C)** Gel images of SAA2 expression in placental explant slices and statistical analysis normalized to the levels of actin as a loading control using Western blot. LPS: 500 ng/ml. **(D)** Cell cytotoxicity was determined by lactate dehydrogenase (LDH) assay. **(E)** Flow cytometry was performed to measure CD45+ leukocytes in the placenta slices. Debris and doublets were excluded by gating on side scatter height SSC-H versus SSC-A to delaminate singlets. Placental leukocytes were gated sequentially on CD45 + versus SSC-A properties. The ratio of CD45+ leukocytes in the placenta slices was statically compared between groups. The concentrations of IL-1β **(F)** and TNF-α **(G)** in the culture media were measured by enzyme-linked immunosorbent assay (ELISA). PBS+PBS, n = 4; LPS+PBS, n = 4; LPS+si*Saa2*, n = 3; PBS+si*Saa2*, n = 3. Values are expressed as mean ± SEM, *p < 0.05, **p < 0.01, ***p < 0.001. One-way ANOVA.

To further confirm the key role of placental SAA2 in mediating intrauterine inflammation-induced PTB, explant culture of placental tissue was performed on E17. Isolated placentas were incubated with varying concentrations of LPS (10, 100, 500, 1,000 ng/ml) with or without si*Saa2* administration. After 24 h, there were no changes in SAA1 expression (**Figure 4A**) while SAA2 expression (**Figure 4B**) was increased in a dose-dependent manner with inflammation. The effect of si*Saa2* was validated by examining the protein level of SAA2 expression using Western blot. SAA2 was significantly decreased by maternally administered si*Saa2* (*p* < 0.05, **Figure 4C**). Effectively, si*Saa2* administration to the inflamed placental explants markedly reduced cell death (*p* < 0.05, **Figure 4D**), decreased CD45+ leucocytes (*p* < 0.05, **Figure 4E**) using flow cytometry, and the release of IL-1β and TNF-α into the media (*p* < 0.05 or 0.001, **Figures 4F, G**) which were increased by LPS treatment. Our data support that SAA2 in the placenta plays an important role in the mechanism of intrauterine inflammation and the subsequent adverse outcomes.

### Maternal administration of siSaa2 reduces proinflammatory cytokine expression and CD45+ leukocyte infiltration induced by intrauterine inflammation in the placenta

**Figure 5:**
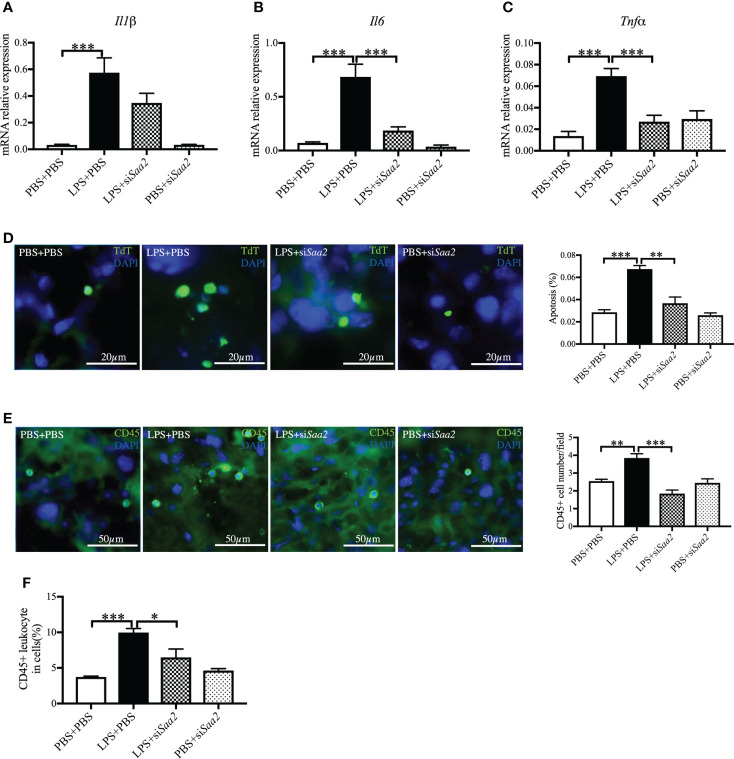
siRNA against *Saa2* inhibits placental *Il6* and *Tnfα* expressions induced by intrauterine inflammation. On embryonic (E) day 17, pregnant CD-1 mice underwent a mini-laparotomy in the lower abdomen for intrauterine injection of lipopolysaccharide (LPS). One hour later, dams received intravenous injection of siRNA against serum amyloid A 2 (si*Saa2*) or phosphate-buffered saline (PBS). At 24 h postinjection (hpi), dams were sacrificed. Placentas were harvested from each dam, and real time-quantitative polymerase chain reaction (RT-qPCR) was performed to evaluate the effect of siRNA treatment. The levels of *Il1β* **(A)**, *Il6* **(B)**, and *Tnfα* **(C)** were statically compared between groups. PBS+PBS, n = 6; LPS+PBS, n = 10; LPS+si*Saa2*, n = 7–9; PBS+si*Saa2*, n = 6. **(D)** Apoptotic rates of placental cells treated as indicated were detected by terminal deoxynucleotidyl transferase dUTP nick end labeling (TUNEL) staining. Representative images for positive cells were labeled by TdT (green) and DAPI (blue, nucleic staining) in the labyrinth of placentas. Scale bars: 20 μm. **(E)** Representative fluorescence immunostaining for CD45+ (green) and DAPI (blue, nucleic staining) in the labyrinth of placentas. Scale bars: 50 μm. **(F)** The ratio of CD45+ leukocytes infiltrated in the placenta were statically compared between groups by flow cytometry. Each group, n = 4. Values are expressed as mean ± SEM, *p < 0.05, **p < 0.01, ***p < 0.001 by one-way ANOVA.

To explore the molecular mechanism, we further measured the expression of pro-inflammatory cytokines, including *Il1β*, *Il6*, and *Tnfα*. The mRNA expressions of *Il1β*, *Il6*, and *Tnfα* in the placenta were significantly increased in LPS+PBS compared with PBS+PBS (*p* < 0.001, ). Maternal administration of si*Saa2* (LPS+si*Saa2*) markedly decreased the expressions of *Il6* and *Tnfα* compared with LPS+PBS (*p* < 0.001, **Figures 5B, C** **)**. There was a decreasing trend of *Il1β* expression but not reaching statistical significance in LPS+si*Saa2* compared with LPS+PBS (**Figure 5A**). Furthermore, maternal administration of si*Saa2* significantly decreased cell death in the placenta (**Figure 5D**), and CD45+ leukocytes infiltrated in the placenta (**Figures 5E, F** **)** in the LPS+si*Saa2* group compared with the LPS+PBS group. These data indicate that inhibition of *Saa2* reduces the immune responses and cell death in the placenta exposed to intrauterine inflammation.

### Maternal administration of siSaa2 does not change Saa1–4 expression in the fetal brain exposed to intrauterine inflammation

**Figure 6:**
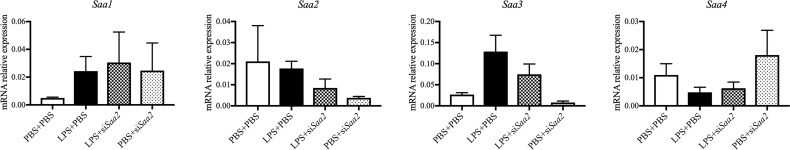
siRNA against *Saa2* does not change *Saa1–4* expression in the fetal brain exposed to intrauterine inflammation. On embryonic (E) day 17, CD-1 mice underwent a mini-laparotomy in the lower abdomen for intrauterine injection of lipopolysaccharide (LPS). One hour later, dams received intravenous injection of siRNA against serum amyloid A 2 (si*Saa2*) or an infusion of phosphate-buffered saline (PBS). At 24 h postinjection (hpi), dams were sacrificed. Fetal brains were harvested from one embryo of each dam. Real time-quantitative polymerase chain reaction (RT-qPCR) on *Saa1–4* was performed to evaluate the effect of maternal siRNA treatment. PBS+PBS, n = 4; LPS+PBS, n = 7; LPS+si*Saa2*, n = 5–6; PBS+si*Saa2*, n = 3–4. Values are expressed as mean ± SEM, one-way ANOVA.

To explore if maternal administration of si*Saa2* alters the expression of Saa isoforms in the fetal brain, which is highly susceptible to maternal infection and inflammation, we compared the expression of fetal *Saa* in the presence of LPS with or without siRNA. RT-qPCR was performed in the fetal brain. There were no significant changes in *Saa1–4* between groups exposed to either intrauterine inflammation or maternal siRNA treatment at 24 hpi (**Figure 6**). Our data imply that fetal *Saa* isoforms do not respond to either intrauterine inflammation or maternal administration of si*Saa2*.

## Discussion

For the first time, our study provides evidence that placental SAA2, but not SAA1, 3, and 4, participates in the mechanism of PTB induced by intrauterine inflammation. In developing placenta, SAA2 expression was absent from the completion of placenta until delivery. Following intrauterine inflammation, placental SAA2 increased while other isoforms of SAA did not. Maternal administration of siRNA against *Saa2* alleviated PTB *via* decreasing the expression of pro-inflammatory cytokines *Il1β*, *Il6*, and *Tnfα*. The expression of *Saa* isoforms in the fetal brain was not affected by maternal inhibition of *Saa2*.

It has been generally assumed that *SAA1* and *SAA2* are coordinately regulated and that their protein products play essentially identical roles (44). Many studies mention either SAA1 only or so-called SAA indistinctly (14, 15, 18, 27, 45). However, the apparent paradox of why higher mammals have at least two SAAs has not been solved thus far. SAA1 can be synthesized in human placenta and increased associated with parturition (28, 46). On the contrary, in a mouse model of PTB, SAA2 levels significantly increase in plasma (47). Our data demonstrated that SAA1 and SAA2 were differentially regulated during development and inflammation in the placenta and maternal liver. Thus, it is indispensable to fully characterize SAAs within species and tissues under a variety of conditions. Understanding the shared and divergent aspects of SAAs may help to aim at devising therapeutic strategies to prevent PTB.

It is suggested that SAA is a multifunctional protein by transitioning between pro-inflammatory and anti-inflammatory functions according to the stage of inflammation (23, 43, 48). In our study, we used SAA1- or SAA2-derived peptides as our stimulators (14). Maternally administered SAA1 or SAA2 only or following inflammation did not affect abortion or PTB, which is different from previous studies (28, 29). These differences may be due to the origin (recombinant *vs*. endogenous), variable dose effects, or animal models applied. Nevertheless, our study including the *in vivo* and explant culture indicates that placental SAA2 is a primary pro-inflammatory factor of the induction of PTB following intrauterine inflammation.

SAA synthesis is regulated by pro-inflammatory cytokines (49, 50). SAA is an endogenous ligand for TLR2, TLR4, and P2X7R (23, 28, 51, 52). In addition, SAA genes contain either a nuclear factor interlukin-6 (NF-IL6) binding site (44) or the CTGGGA sequence, which is typically present in the promotor region of acute-phase proteins (APR) (53). By these SAA-mediated non-specific bindings, more inflammatory cytokines and APRs are generated to advance the immune responses. Studies have shown that IL-1β, IL-6, and TNF-α are the major pro-inflammatory cytokines associated with PTB (54, 55). IL-6 is produced at the site of inflammation and is a chief stimulator of the production of most acute-phase proteins (56). In the pregnant rhesus monkeys exposed to bacteria, only TNF-α and IL-1β are capable of provoking intense uterine contractions resulting ultimately in PTB (57, 58). In our study, inflammation significantly increased the expressions of pro-inflammatory cytokines including *Il6*, *Tnfα*, and *Il1β*. Inhibition of *Saa2* decreased the expressions of *Il6*, *Tnfα*, and *Il1β*. Maternal administration of siRNA against *Saa2* induces the degradation of *Saa2* and decreases the transcription of SAA2, ultimately inhibiting the production of pro-inflammatory cytokines. Our study indicates the mechanism that si*Saa2* to alleviate PTB involves the downregulation of pro-inflammatory cytokines.

Although mRNA levels of all three gene isoforms *Saa1–3* significantly increased in the placenta, only SAA2 was elevated at the protein level. Similar results have been reported in other studies (59, 60). Thus, we hypothesize that regulation of mRNA stabilization (61) and/or posttranscriptional modifications (62, 63) likely contributes to production of varying SAA1 and SAA3 proteins. Future research should be conducted to resolve these differences across different cells, tissues, and organisms.

CD45 is usually applied as a leukocyte common antigen biomarker (64, 65). Similar to another study (66), the elevated number of CD45+ cells induced by LPS was observed in our explant culture of placenta. Inhibition of *Saa2* decreased the CD45+ cells. Hofbauer cells are fetal placental macrophages and the primary leukocytes in the placenta (67, 68). Hofbauer cells have a stem-cell-like phenotype and can undergo proliferation, expanding in numbers following infection (69–71). In our study, the increased CD45+ cells in LPS-treated explant may be due to the increased proliferation of Hofbauer cells. si*Saa2* treatment may decrease the proliferation of Hofbauer cells. Studies have demonstrated that SAA is capable of inducing differentiation and proliferation of many cell types including macrophages (72–74). Since CD45+ hematopoietic stem cells in the placenta are very few or no more are found after E15.5 (75), our explant culture of placental tissue provides a possible mechanism that si*Saa2* alleviated LPS-induced inflammation through the inhibition of proliferation of Hofbauer cells.

The most clinically advanced siRNA technology (animal free-origin lipid nanoparticle formula) especially favors tissues with high blood flow (76), which facilitates the vascular placenta to be a highly selective organ for siRNA therapy. In our study, the expression of placenta SAA2 only occurred with inflammation. Maternal administration of corresponding siRNA to *Saa2* effectively and selectively reduced SAA2 expression in the placenta, with much less effect in the maternal liver. Targeting placental SAA2, but not other mediators which are also expressed under physiological conditions besides inflammation, may avoid potential interference from widespread side effects or ineffectiveness.

We found that maternal infusion of si*Saa2* did not interfere with the expression of fetal SAAs. These findings, therefore, minimize the concerns that potential SAA silencing in the fetus would negatively affect fetal development and physiology (76). Other studies also confirmed that siRNA administered during pregnancy is not transmitted to fetal tissues other than the placenta due to their relatively large and negatively charged molecular features (77). The higher specificity in gene silencing effect makes siRNA to be more considered in clinical applications, although efficient and safe delivery into target cells remains a significant challenge that needs to be resolved. Our future study will further evaluate the effect of delivery routes and doses on the safety of fetus in the long term.

In conclusion, our study highlights the variable expression patterns of placental SAA isoforms during gestational course and intrauterine inflammation-associated PTB. Placental SAA2 plays a vital role in the mechanism of PTB induction *via* regulating the expression of pro-inflammatory cytokines. Maternal siRNA treatment opens a new avenue for the therapy of PTB induced by intrauterine inflammation.

## Data availability statement

The original contributions presented in the study are included in the article/**Supplementary Material**. Further inquiries can be directed to the corresponding authors.

## Ethics statement

This study was reviewed and approved by The Institutional Animal Care and Use Committee and Health Safety and the Environment at Johns Hopkins University.

## Author contributions

JunL, YL and IB conceived of the study and experimental questions, YL, JunL, AL, HY and JinL collected and provided samples, YL, JunL and JinL processed samples, YL and JunL analyzed and graphed data, JunL, YL and IB wrote the manuscript. All authors contributed to the article and approved the submitted version.

## Acknowledgments

This work was supported by Johns Hopkins Intergraded Center for Fetal Medicine (IB) and Sheikh Abdullah Bugshan Fund (IB).

## Conflict of interest

The authors declare that the research was conducted in the absence of any commercial or financial relationships that could be construed as a potential conflict of interest.

## Publisher’s note

All claims expressed in this article are solely those of the authors and do not necessarily represent those of their affiliated organizations, or those of the publisher, the editors and the reviewers. Any product that may be evaluated in this article, or claim that may be made by its manufacturer, is not guaranteed or endorsed by the publisher.
