# Supplementary material for: Maternal siRNA silencing of placental SAA2 mitigates preterm birth following intrauterine inflammation
Source: Front Immunol. 2022 Sep 23;13:902096. doi: 10.3389/fimmu.2022.902096 (PMC9539923; doi:10.3389/fimmu.2022.902096)
Supplement: Supplementary file 1 [file DataSheet_1.pdf]

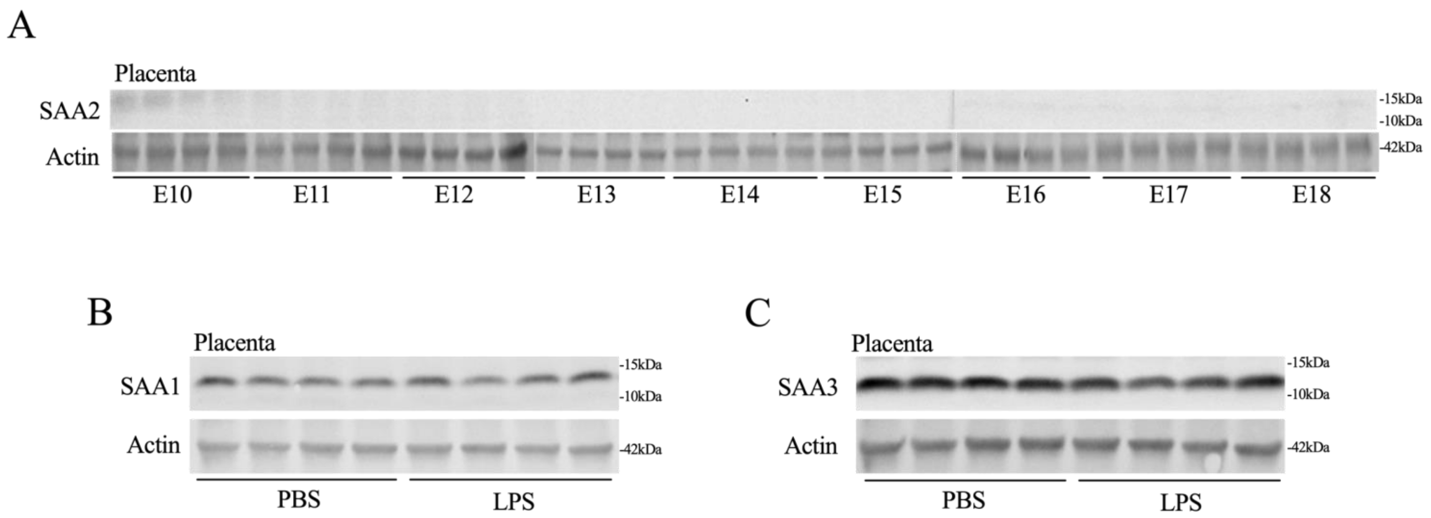

Supplement Figure 1. SAA1, SAA2 and SAA3 from different commercially provided antibodies. (A) One placenta was harvested daily from each CD-1 dam starting from embryonic (E) day 10 and ending on E18. Western blot was performed to detect the expression of SAA2 protein (LSBio). **(B-C)** On E17, CD-1 mice underwent a mini-laparotomy in the lower abdomen for intrauterine injection of lipopolysaccharide (LPS). Placenta were harvested within the injected uterine horn that were viable at 24 hours post injection (hpi). Western blot was performed to detect the expression of SAA1 protein (LSBio) **(B)** and SAA3 protein (Abcam) **(C)**.

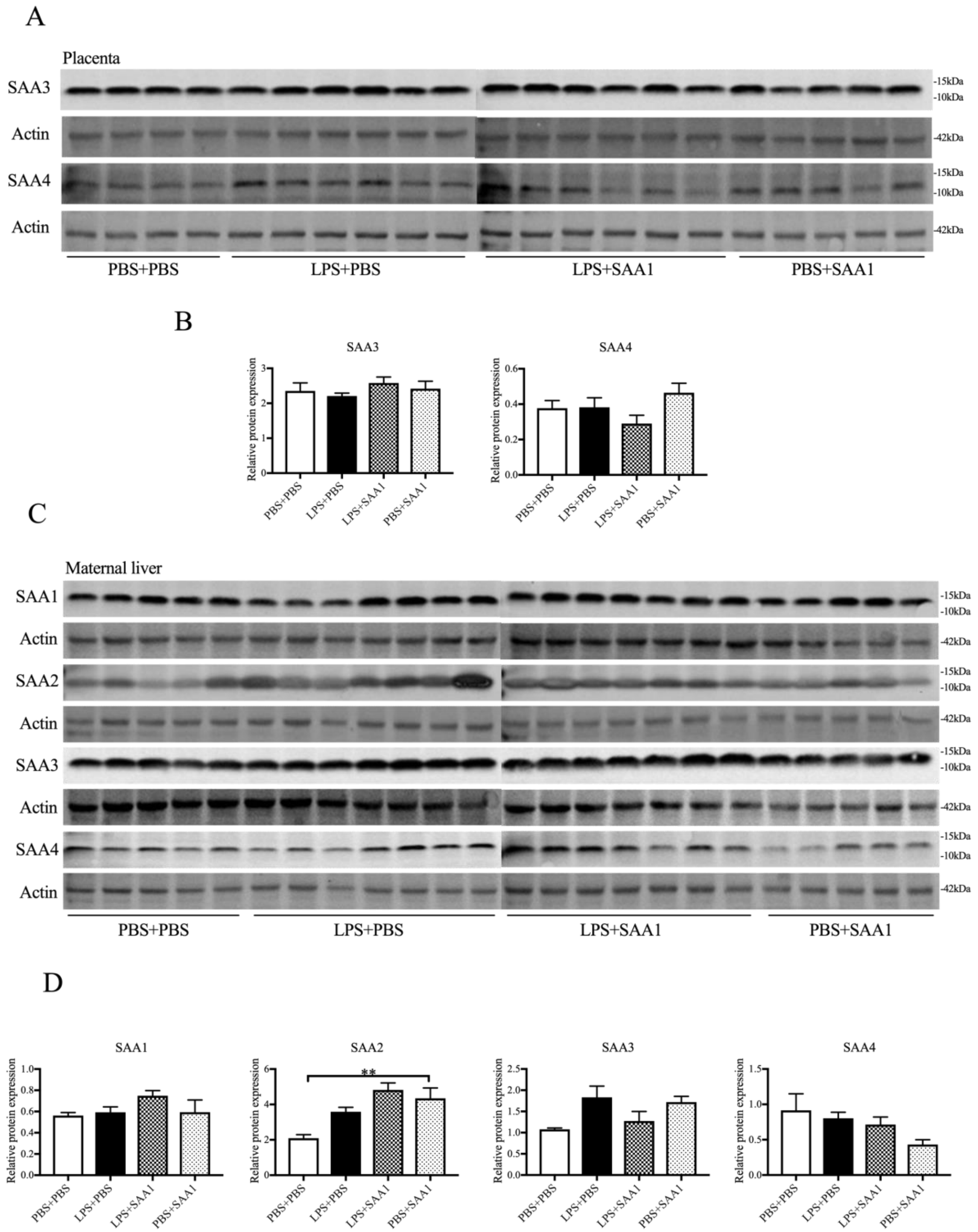

Supplement Figure 2. The expression of serum amyloid A (SAA) in the placenta and maternal liver following intrauterine inflammation. On embryonic (E) day 17, CD-1 mice underwent a mini-laparotomy in the lower abdomen for intrauterine injection of lipopolysaccharide (LPS). One hour later, dams received intraperitoneal injection of SAA1 fragment or phosphate-buffered saline (PBS). At 24 hour post injection (hpi) dams were sacrificed. Placentas and maternal livers were harvested from one embryo of each dam. **(A)-(B)** Gel images of SAA3 and SAA4 in the placenta and statistical analysis normalized to the levels of Actin as a loading control with SAA1 injection. PBS+PBS, n=4; LPS+PBS, n=6; LPS+SAA1, n=6; PBS+SAA1, n=5. **(C)-(D)** Gel images of SAA1-4 expressions in the maternal liver and statistical analysis normalized to the levels of Actin as a loading control with SAA1 injection. PBS+PBS, n=5; LPS+PBS, n=7; LPS+SAA1, n=7; PBS+SAA1, n=5. Values are expressed as mean±SEM, $^{**}p<0.01$ by One-Way ANOVA.

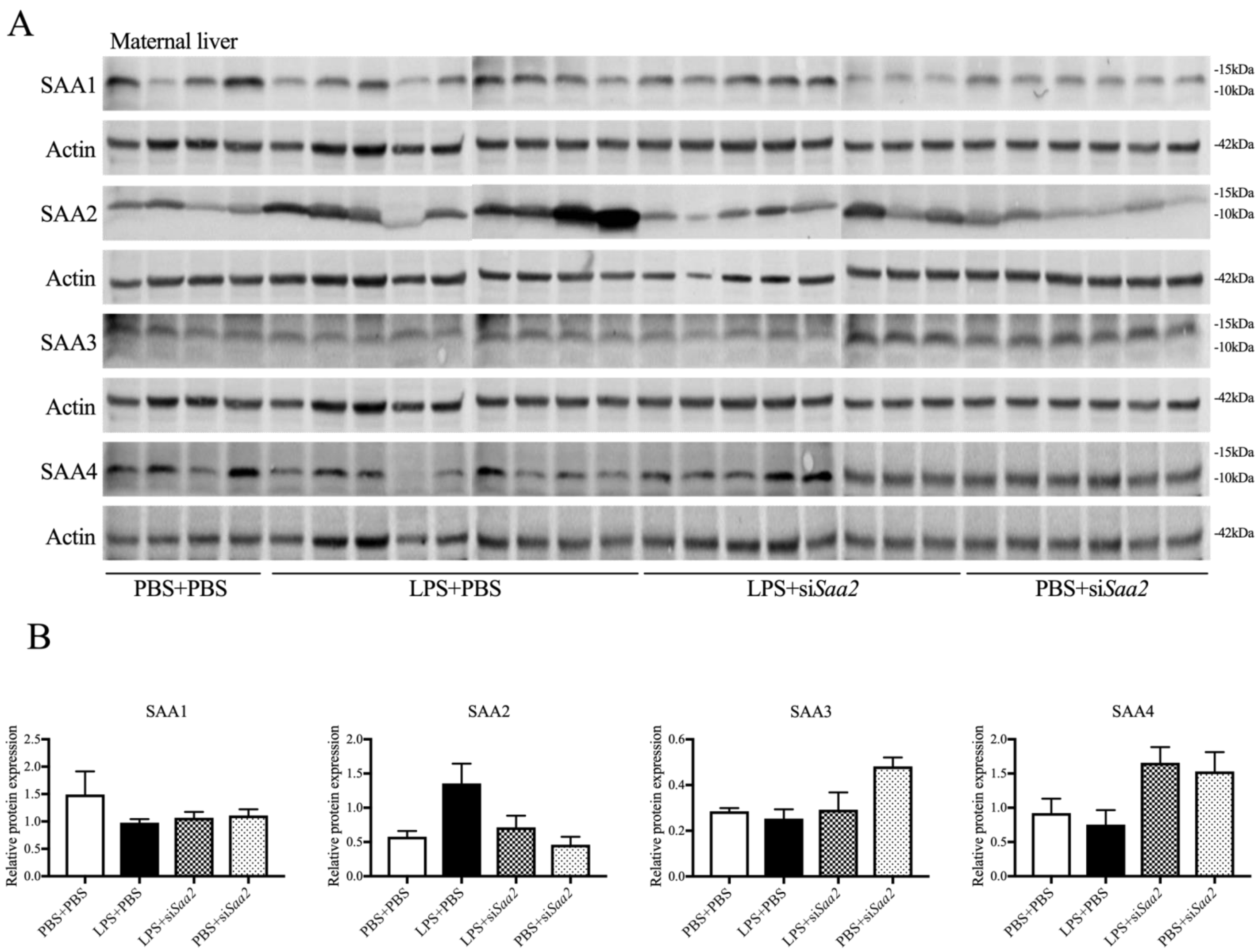

Supplemental Figure 3. The expression of serum amyloid A (SAA) in the maternal liver following intrauterine inflammation and maternal administration of siRNA against *Saa2*. On embryonic (E) day 17, CD-1 mice underwent a mini-laparotomy in the lower abdomen for intrauterine injection of lipopolysaccharide (LPS). One hour later, dams received intravenous injection of siRNA against *Saa2* (si*Saa2*) or phosphate-buffered saline (PBS). At 24 hour post injection (hpi) dams were sacrificed. Maternal livers were harvested from each dam. **(A)-(B)** Gel images of SAA1-4 expressions in the maternal liver and statistical analysis normalized to the levels of Actin as a loading control. PBS+PBS, n=4; LPS+PBS, n=7; LPS+si*Saa2*, n=8; PBS+ si*Saa2*, n=6. Values are expressed as mean±SEM, by One-Way ANOVA
